# Blood pressure measurements for diagnosing hypertension in primary care: room for improvement

**DOI:** 10.1186/s12875-023-02241-z

**Published:** 2024-01-02

**Authors:** Vincent M.I. Voorbrood, Evelien I.T. de Schepper, Arthur M. Bohnen, Marit F.E. Ruiterkamp, Peter R. Rijnbeek, Patrick J.E. Bindels

**Affiliations:** 1 Department of General Practice, Erasmus MC, P.O. Box 2040, Rotterdam, 3000CA the Netherlands; 2 Department of Medical Informatics, Erasmus MC, Rotterdam, the Netherlands

**Keywords:** Primary care, Hypertension, Diagnosis of Hypertension

## Abstract

**Background:**

In the adult population, about 50% have hypertension, a risk factor for cardiovascular disease and subsequent premature death. Little is known about the quality of the methods used to diagnose hypertension in primary care.

**Objectives:**

The objective was to assess the frequency of use of recognized methods to establish a diagnosis of hypertension, and specifically for OBPM, whether three distinct measurements were taken, and how correctly the blood pressure levels were interpreted.

**Methods:**

A retrospective population-based cohort study using electronic medical records of patients aged between 40 and 70 years, who visited their general practitioner (GP) with a new-onset of hypertension in the years 2012, 2016, 2019, and 2020. A visual chart review of the electronic medical records was used to assess the methods employed to diagnose hypertension in a random sample of 500 patients. The blood pressure measurement method was considered complete if three or more valid office blood pressure measurements (OBPM) were performed, or home-based blood pressure measurements (HBPM), the office- based 30-minute method (OBP30), or 24-hour ambulatory blood pressure measurements (24 H-ABPM) were used.

**Results:**

In all study years, OBPM was the most frequently used method to diagnose new-onset hypertension in patients. The OBP-30 method was used in 0.4% (2012), 4.2% (2016), 10.6% (2019), and 9.8% (2020) of patients respectively, 24 H-ABPM in 16.0%, 22.2%, 17.2%, and 19.0% of patients and HBPM measurements in 5.4%, 8.4%, 7.6%, and 7.8% of patients, respectively. A diagnosis of hypertension based on only one or two office measurements occurred in 85.2% (2012), 87.9% (2016), 94.4% (2019), and 96.8% (2020) of all patients with OBPM. In cases of incomplete measurement and incorrect interpretation, medication was still started in 64% of cases in 2012, 56% (2016), 60% (2019), and 73% (2020).

**Conclusion:**

OBPM is still the most often used method to diagnose hypertension in primary care. The diagnosis was often incomplete or misinterpreted using incorrect cut-off levels. A small improvement occurred between 2012 and 2016 but no further progress was seen in 2019 or 2020. If hypertension is inappropriately diagnosed, it may result in under treatment or in prolonged, unnecessary treatment of patients. There is room for improvement in the general practice setting.

**Supplementary Information:**

The online version contains supplementary material available at 10.1186/s12875-023-02241-z.

## Introduction

Approximately 50% of the adult population aged between 40 and 70 years has been diagnosed with hypertension [1, 2]. Systolic hypertension is the most preventable cause of strokes, myocardial infarction, heart failure, chronic kidney disease, and premature death [2]. In 2017, cardiovascular diseases (CVD) were the second most common cause of death in the Netherlands with arterial hypertension being a common risk factor [2–6]. Therefore, it is important to normalize systolic blood pressure (SBP) to physiological values. Several cardiovascular risk-management tools were developed to detect, treat, and follow-up on risk factors for CVD [5–10].

Important treatment elements include diminishing SBP, cessation of smoking, weight loss, lowering blood lipids, and promoting physical activity.

In the Netherlands, the preferred way to diagnose hypertension is described in a guideline by the Dutch College of General Practitioners (NHG), and this guidelines was revised in 2012 [11, 12].

However, in recent years, there has been more emphasis on alternative methods of blood pressure measurement [5]. A number of methods are recommended to detect hypertension, these include office blood pressure measurements (OBPM), automatic office blood pressure monitoring for 30 min (OBP30), ambulatory home blood pressure measurements (HBPM), and 24-hour ambulatory blood pressure measurements (24 H-ABPM) [13–18].

Applying a correct method to measure and a proper cut-off value is essential to prevent unnecessary use of lifelong medication with yearly follow-up by blood sample investigation and SBP measurements.

Nevertheless, potential biases can surface from blood pressure measurements, which each method is known to have both its advantages and limitations [5, 7]. For instance, SBP is known to have temporal variations, necessitating multiple measurements over several months in order to obtain a valid diagnosis which in turn is needed to make decisions regarding whether to initiate long-term drug therapy [5]. OBPM may also introduce biases, including white coat hypertension (WCH) and “masked” hypertension [19, 20]. That is why the NHG to recommends in case of an elevated blood pressure to do multiple OBP measurements on different days [11]. The European Health Community discourages relying solely on sphygmomanometer to record SBP and advocates for the broader utilization of out-of-office BP measurements like 24 H-ABPM or HBPM [5, 15, 16]. Despite the importance of accurate diagnosis, there remains limited research on the quality of the methods used to diagnose hypertension in general practice. Older reports suggest that hypertension is often incorrectly diagnosed [21, 22].

The primary aim of this study was to assess the frequency of use of recognized methods to establish a diagnosis of hypertension and specifically, for OBPM whether three distinct measurements were taken, and how correctly the blood pressure levels were interpreted. The secondary aim was to determine how often inappropriate diagnoses were followed by the start of medication. Finally, we were interested in whether the revision of the guidelines in 2012 influenced the method and interpretation of blood pressure measurements in the general practice setting.

## Methods

### The IPCI database

A population based cohort study was performed using the Integrated Primary Care Information (IPCI) database of the Department of Medical Informatics, Erasmus Medical Center, Rotterdam.

IPCI is a database containing pseudonymised longitudinal medical data from general practice on demographics, symptoms, and diagnosis, as well as correspondences with secondary care. It contains data of 2.7 million patients and is considered representative of the whole Dutch population [23]. This study was approved on 7 November 2017 by the “Raad van Toezicht van IPCI” (IPCI Review Board project number: 9/2017). The board waived the requirement for informed consent as the study involved analysis of pseudonymised medical records without direct contact with patients. All methods were carried out in accordance with relevant guidelines and regulations.

It is obligatory for Dutch citizens to be registered with a single general practitioner (GP). The GP is the first point of contact for health-related questions and medical care (gatekeeper). Medical diagnoses are recorded using the International Classification of Primary Care (ICPC) codes [24] while drug-related data are recorded using the Anatomical Therapeutic Chemical (ATC) classification system. Other clinical information, such as blood pressure measurements, are recorded as measurement codes or as free text.

### Selection of patients

In the IPCI database, we selected patients between the ages of 40 and 70 who visited their GP in the study years 2012, 2016, 2019, and the second half of 2020. The age group of 40 and 70 years was chosen because a new diagnosis of hypertension is usually present after the age of 40. Comorbidity is most prevalent above the age of 70. These patients were diagnosed with first onset hypertension. The medical diagnosis was extracted using the ICPC codes for uncomplicated hypertension (K86) and complicated hypertension (K87). The year 2012 was chosen since an updated guideline for the diagnosis of hypertension was published in that year [12]. The year 2016 was chosen because it was four years after the introduction of this guideline. We assumed that GPs would need 3–4 years to adapt to new developments and implement the guidelines in practice. The years 2019 and 2020 were chosen as follow-up years. The first half of 2020 was not taken into account in our analysis due to the start of COVID-19 pandemic (lock down).

**Fig. 1:**
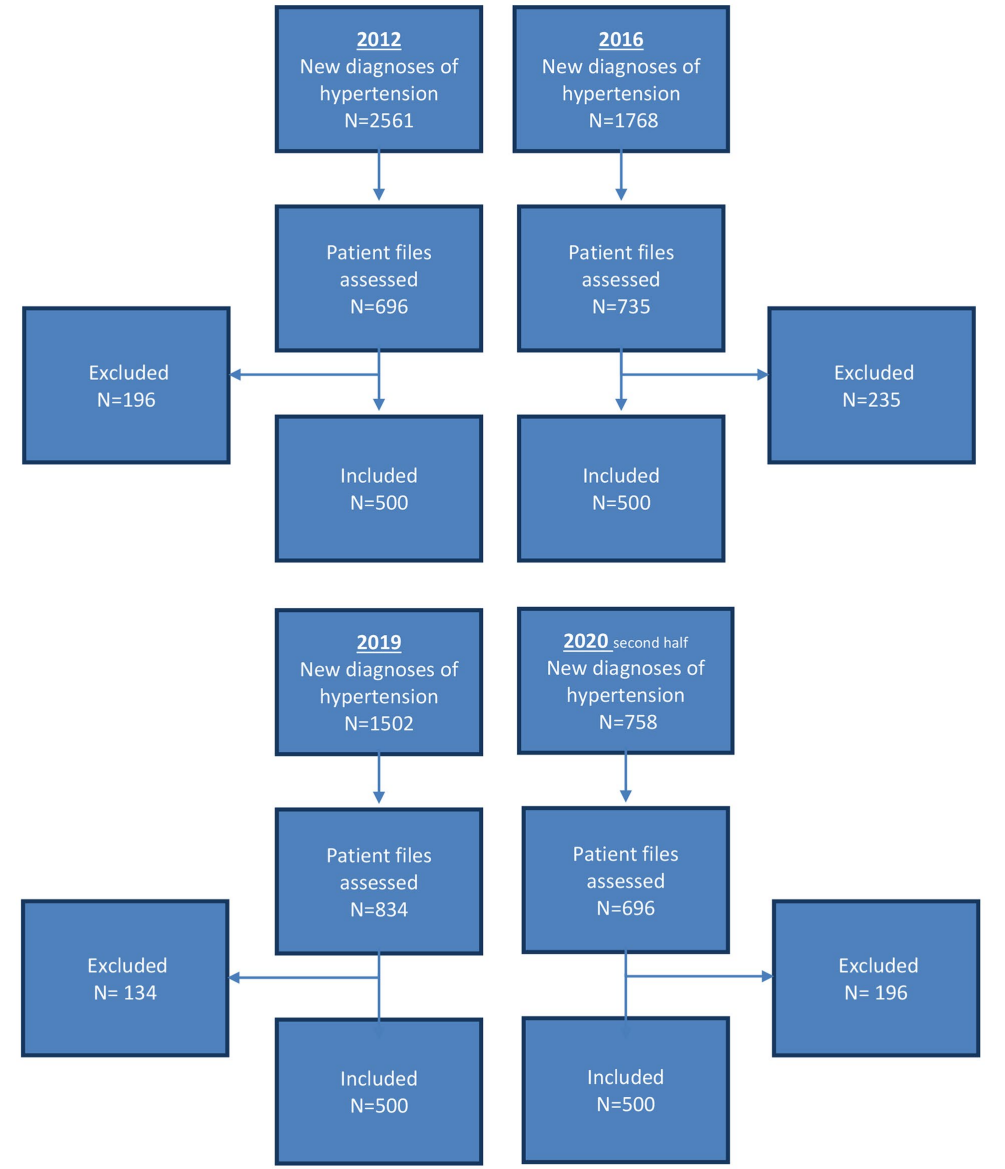
Flowchart with new diagnosis assessed patient files and exclusions

We included patients of GP practices with valid data in the IPCI database during all study years. Patients were excluded first if they had less than 6 months of follow-up in the IPCI database after the date of the first diagnosis of hypertension. Second patients with a pre-existing diagnosis of a cardiovascular event (e.g., myocardial infarction, cerebral vascular accident), ICPC K75, K76.01, K89, K90.02, and K90.03). Third if hypertension had not been diagnosed in general practice but in another setting (e.g. hospital). The next reason for exclusion was the lack of blood pressure readings in the medical records (Fig. 1). Patients taking medication that affected SBP prior to the first date of diagnosis of hypertension were marked in the text required for visual inspection. Prescribed medications were identified by the corresponding ATC codes (CO7A ß- blocking agents, C03 diuretics, C09A angiotensin-converting enzyme inhibitor, C08CA calcium channel blockers, and C09CA angiotensin receptor blocker).

The electronic medical records of the selected patients in each year were randomly selected and all charts were visually reviewed, including the free text. The most frequent reason for exclusion by visual inspection was the prior use of medication (as listed above). For example, ß- blocking agents are also used in patients with migraine. We included up to 500 study patients per year who were eligible for analysis.

### Method of SBP measurements

The method used to measure BP, the dates of measurements, and the SBP were recorded up to six months prior to the first diagnosis of hypertension. The date of the first diagnosis was recorded. The start date of antihypertensive medication was also recorded after the diagnosis of hypertension (within half a year).

Diagnostic methods were categorized as OBPM, OBP30, 24 H-ABPM method, or HBPM.

BP measurements were defined as complete if one of the following methods was used: OBPM with a mean blood pressure of at least three related measurements on different days. In the HBPM, OBP30, and 24 H-ABPM, the number of measurements was not assessed; reporting of a mean systolic blood pressure value was required. If multiple methods were used for a given patient within a period of 3 months, the best quality method was chosen. For instance, if there was a single office measurement followed later on by an OBP30, HBPM, or 24 H-ABPM, the latter was chosen.

### Interpretation of SBP readings

In addition to the completeness of the procedure, the correct interpretation of the measurements was assessed. The mean SBP cut-off values for diagnosing hypertension were defined for different types of SBP measurements (Supplementary Table 1) [5, 6]. The method of diagnosis was defined as correct if both the measurement method and the interpretation of the SBP value (right cut-off values) were correct. In OBPM, OBP30, and HBPM, the mean SBP was evaluated. In the 24 H-ABPM method, the SBP was interpreted and judged correctly if the mean daytime SBP was above the cut-off. In the SBP values different cut-off values were selected for interpretation (Table 3) (VV reviewed all charts). To check for accuracy, two authors (VV and MR) independently reviewed 240 medical files in the years 2012 and 2016. Correct diagnoses of hypertension between the VV and MR were found in 15.2% vs. 14% and 25.2% vs. 28%, respectively (r = 0.99).

### Statistical analysis

The final sample exceeded the pre-calculated minimum sample size of 350 patients per year (based on a power of 80% and α of 0.05% to detect an increase from 30% correct diagnoses in one year to 40% in another year) to allow for subgroup analyses. Comparisons between the years were made using chi-square tests. IBM SPSS Statistics 28 was used for the statistical analysis.

## Results

### Baseline characteristics

The sample consisted of 2000 records of patients with newly diagnosed hypertension; 500 records per study year (Fig. 1). In the age category 60–70 years there was a decrease in the percentage of patients diagnosed with hypertension between 2016 and 2020 (43.2% vs. 33.4%, p < 0.05). Compared to females, males were most commonly diagnosed with hypertension in 2016, 2019, and 2020 (Table 1).

**Table 1:** Characteristics of patients (N = 500 per year) and method of diagnosis of hypertension in general practice in 2012, 2016, 2019 and 2020

	Year			
	2012	2016	2019	2020
	N (%)	N (%)	N (%)	N (%)
**Age (years)**				
40–50	124 (24.8)	92 (18.4)	118 (23.6)	110 (22.0)
50–60	177 (35.4)	192 (38.4)	188 (37.6)	223 (44.6) *
60–70	199 (39.8)	216 (43.2)	194 (38.8)	167 (33.4)***
**Gender**				
Male	239 (47.8)	252 (50.4)	258 (51.6)	270 (54.0)
**Method**				
24 H-ABPM	80 (16.0)	111 (22.2)	86 (17.2)	95 (19.0)
OBP30	2 (0.4)	21 (4.2)*	53 (10.6) **	49 (9.8)**
HBPM	27 (5.4)	42 (8.4)	38 (7.6)	39 (7.8)
**OBPM (Number of measurements)**				
1	188 (51.5)	176 (54.5)	218 (67.5)**	194 (61.4)
2	123 (33.7)	108 (33.4)	87 (26.9)	112 (35.4)
3	43 (11.8)	25 (7.7)	13 (4.0)*	6 (1.9 )**
4	9 (2.5)	10 (3.1)	5 (1.5)	3 (0.9)
5	1 (0.3)	3 (0.9)	0 (0.0)	1 (0.3)
7	1 (0.3)	1 (0.3)	0 (0.0)	0 (0.0)
Total	365 (73.0)	323 (64.6)*	323 (64.6)*	316 (63.2)*
**Not determined**	26 (5.2)	3 (0.6)	0 (0.0)	1 (0.2)*

* *p* < 0.05 compared to 2012

**p < 0.05 compared to 2012 and 2016

***p < 0.05 compared to 2016

### Completeness and method of measurements

OBPM was used over the years in 73.0%, 64.6%, 64.6% and 63.2% of the 500 patients in each year, respectively (Table 1). HBPM use slightly increased from 5.4% (2012) to 7.8% (2020). The OBP30 method was significantly more frequently used after 2012 and ranged from 0.4% in 2012 to 10.6% in 2019.

In general, there was a slight, but not statistically significant increase in the use of 24 H-ABPM after 2012. In 2012, in the 365 patients in whom an OBPM was performed, one or two office measurements as an indication for hypertension were found for 311 patients in 2012 (85.2%), 284 of 323 patients in 2016 (87.9%), 305 patients in 2019 (94.4%), and 306 patients in 2020 (96.8%).

A complete OBPM method (three or more measurements) for diagnosing hypertension was performed in 54 of 365 patients (14.9%), 39 patients (12.0%), 18 patients (5.5%) and 10 patients (3.1%) in 2012, 2016, and 2019, and 2020, respectively.

### Correctness of diagnosis

In patients with an OBPM, a correct interpretation was performed in 53%, 50%, 35%, and 42% of patients in 2012, 2016, 2019 and 2020 respectively (p < 0.05 compared to 2012 and 2016) (Table 2).

**Table 2:** Blood pressure measurements and interpretation (n) per method according to the Dutch national guidelines

	Year							
	2012		2016		2019		2020	
Method used	Correctly interpreted (n) total (N)	Correctly interpreted (%)	Correctly interpreted (n) total (N)	Correctly interpreted (%)	Correctly interpreted (n) total (N)	Correctly interpreted (%)	Correctly interpreted (n) total (N)	Correctly interpreted (%)
24 H-ABPM	66/80	83	94/111	85	79/86	92	89/95	94
OBP30	2/2	100	19/21	91	51/53	96	47/49	96
HBPM	27/27	100	42/42	100	38/38	100	37/39	95
OBPM	193/365	53	162/323	50	114/323	35**	134/316	42*

*p < 0.05 compared to 2012 and 2016

**p < 0.05 compared to 2012

In patients with a HBPM, this was 100% (2012), 100% (2016), 100% (2019), and 95% in 2020.

For patients in whom OBP30 was performed, 100% (2012), 91% (2016), 96% (2019), and 96% (2020) had the correct method and interpretation.

In patients with a correctly used 24 H-ABPM method, 66 of 80 (83%) also had a correct interpretation of the cut-off value in 2012, 94 of 11 patients (85%) in 2016, 79 of 86 (92%) in 2019, and 89 (94%) in 2020.

The highest proportion of complete methods and correct interpretations was found in the SBP range 130–149 mm Hg (74%) in 2016, and the highest proportion of correct diagnosis was also found in the SBP range 150–169 mm Hg and 170–199 mm Hg (67%) in 2012 and 59% in 2016, respectively (Table 3).

**Table 3:** Blood pressure measurements and interpretation conform guideline by mean SBP

	Year							
	2012		2016		2019		2020	
SBP mmHg	Correctly interpreted (n) total (N)	Correctly interpreted (%)	Correctly interpreted (n) total (N)	Correctly interpreted (%)	Correctly interpreted (n) total (N)	Correctly interpreted (%)	Correctly interpreted (n) total (N)	Correctly interpreted (%)
< 130	0/14	0	0/15	0	0/14	0	0/14	0
130–149	55/84	66	76/103	74	63/101	62	58/139	30
150–169	130/193	67	155/235	66	141/233	61	74/209	54
170–199	75/144	52	63/107	59	62/136	46	62/136	46
> 200	29/29	100	24/24	100	26/26	100	28/28	100

### Correctness of method of diagnosis and start of medication

Patients with hypertension and incomplete SBP measurements or incorrect interpretation received antihypertensive medication in n = 136 (64%) in 2012, n = 102 (56%) in 2016, n = 130 (60%) in 2019 and n = 140 (73%) in 2020 received antihypertensive medication. The 2016% is significantly (p˂0.05) less than in 2012, in 2020 compared to 2016 and 2019 (p < 0.05) (Table 4).

**Table 4:** Patients starting antihypertension medication in correct and non-correct diagnosis

	Year			
	2012	2016	2019	2020
Diagnosis according to guideline	N (%)	N (%)	N (%)	N (%)
No	136 (64)	102 (56)	130 (60)	140 (73)*
Yes	236 (82)	231 (73)**	204 (72)**	234 (76)

*p < 0.05 compared to 2016 and 2019

**p < 0.05 compared to 2012

## Discussion

In this retrospective cohort study in a primary care setting, we randomly selected and reviewed charts for 500 patients with a first diagnostic ICPC code for hypertension in 2012, 2016, 2019, and the second half of 2020. In all study years, the most commonly used method was OBPM. One or two BP measurements were performed in the majority of patients in whom OBPM was performed, indicating that in the majority of patients the measurements were incomplete and the diagnosis of hypertension was not performed in accordance with the guidelines. However, the clinical significance of an incomplete OBPM is largely unknown. Poor OBPM accuracy could result in unnecessary treatment and exposure to adverse effects.

Biases from specific methods of SBP measurement in hypertension are extensively described in the literature [21, 22]. The present study is the first of its kind and size to examine the methods used in diagnosing hypertension in primary care and is a representation of a nationwide sample. Furthermore it was not clear from the medical records which devices were used in the OBPM.

A limitation of this study was that only SBP was studied. However, epidemiological studies have provided evidence that if the SBP increases, the risk of stroke or other CVDs also increases [7]. Furthermore, the Dutch guidelines uses SBP for the diagnosis of hypertension [11, 12]. Since establishing a diagnosis based on SBP is a basic step in BP management, this was also the focus of this study. Another limitation of this retrospective study is that it depends largely on correct registration by the GP in the electronic medical record (EMR). We assume that of the measurements taken on multiple days, there is at least one recorded SBP per day. However, if measurements are missing in the EMR, it could mean that we are overestimating the number of incomplete hypertension diagnoses and the percentage of incorrectly started medication.

In HBPM, OBP30, and 24 H-ABPM, the number of measurements was not assessed, and reporting of a mean systolic blood pressure value was required only, yet mistakes are possible. We assume that the GPs who apply these methods are generally well motivated to register the correct measurements in the medical file for the diagnosis of hypertension. It is striking that the interpretation of HBPM was always correct.

It is important for the GP to realize that repeated SBP measurements are required over a longer period of time and are necessary in order to make the correct treatment decision. The 24 H-ABPM and HBPM are necessary to detect white coat hypertension and “masked hypertension“. OBPM shows poor sensitivity and specificity compared to HBPM and 24H-ABPM [25].

HBPM and 24 H-ABPM have consistently been shown to be more sensitive risk predictors than OBPM for outcomes such as coronary morbidity, stroke, or fatal cardiovascular events [6].

It is remarkable that the GPs in our cohort study opted so infrequently for the HBPM, OBP30, or 24 H-ABPM methods. Maybe, there are also barriers to the adoption of the different measurements methods, the HBPM, OBP30 and the 24 H-ABPM require a special device. Even if these methods were chosen, we observed that the interpretation, using the advised cut-off values, was not always correct, especially for 24 H-ABPM measurements. (Table 3).

However, an incomplete measurement method or incorrect interpretation when diagnosing hypertension does not mean that no hypertension would have been found if a complete measurement method and correct interpretation had been applied. This limits the assessment of the overall quality of hypertension management, in which various other factors are important. Firstly, only three indicators were used for assessing quality: the measurement method, the number of measurements, and the use of proper cut-off values before establishing the diagnosis. As this was a review of electronic charts, the quality of the execution of the measurements could not be properly assessed.

Secondly, the decision to start pharmacological treatment depends not only on the SBP level but also on the total cardiovascular risk, which calls for a proper history, physical examination, and laboratory examination. Thirdly, we did not study if the selected type of medication was appropriate, or if treatment goals were reached.

In the second half of 2020, during the COVID-19 pandemic, a change to out of office methods to diagnose hypertension was expected due to possibly increased use of HBPM, OBP30, and 24 H-ABPM because the possibility for office GP consultations was limited due to lockdown strategies [26–28]. Physical consultation were limited. However, changes in these methods could not be observed.

The prevalence of hypertension, using the correct measurement method, seems to be lower if white coat hypertension is excluded [29]. Since the current practice is mainly based on limited office blood-pressure measurements, we question if the prevalence rates of approximately 50% in the age category 35–70 years, based on only an incomplete number of measurements per patient, as currently reported in the literature in the Netherlands, are not an overestimation [1, 12].

If hypertension is correctly diagnosed, in the absence of WCH, the number of hypertensive patients may be lower.

To increase the accuracy and quality of hypertension diagnosis, GPs should actually receive additional support, for instance, a notification in the EMR system of the patient. Such a notification will alert the GP to which SBP method they can best use to diagnose hypertension. We observed that the GP quickly made a diagnosis during the first measurement. A too early start with medication is lurking. An explanation may be that the GP is too concerned about the elevated blood pressure.

The observed high rate of medication started in the incompletely diagnosed patients is a matter of serious concern.

## Conclusion

In our study OBPM was the dominant method for diagnosing hypertension in patients who visited their GP for BPM. Through study years only a limited change to other, more robust, methods was observed. Screening for hypertension using OBPM has major limitations. This study showed that in general practice in the Netherlands, the recommendations in the Dutch and European guidelines were only partially followed. A small improvement occurred between 2012 and 2016. No progress was seen in the years 2019 and 2020. Despite an incomplete method of diagnosis, medication was prescribed to the majority of patients.

Patients benefit from a correct diagnosis and treatment because the risk of CVD complications can be lowered. An incorrect start of treatment may mean unnecessary lifelong use of medication and follow-up. The observed high rate of medication started in the incomplete diagnosed patients is a matter of serious concern .There is still an urgent need for increased awareness and actual change in primary care to properly use methods of blood pressure measurement. Unfortunately, there is still much room for improvement.

### Electronic supplementary material

Below is the link to the electronic supplementary material.

Supplementary Material 1

## Data Availability

The data and materials are not freely accessible, because IPCI is an anonymized database. The data that support the findings of this study are available from the IPCI database but restrictions apply to the availability of these data, which were used under license for the current study, and so are not publicly available. Data are however available from the authors upon reasonable request and with permission of the IPCI Review board. The authors only have permission to share metadata with third parties. Metadata is referred as descriptive variable information. As study data was pseudonymised, it is not possible to obtain nor share identifiable information. Extensive measures are in place to ensure confidentiality and ethical conduct of the research. The corresponding author V.M.I. Voorbrood can be contacted to access data.
