# Supplementary material for: Blood pressure measurements for diagnosing hypertension in primary care: room for improvement
Source: BMC Prim Care. 2024 Jan 2;25:6. doi: 10.1186/s12875-023-02241-z (PMC10759563; doi:10.1186/s12875-023-02241-z)
Supplement: Supplementary file 1 — Supplementary Material 1 [file 12875_2023_2241_MOESM1_ESM.docx]

**Supplementary**

Supplementary Table 1. Cut-off values for diagnosing hypertension

| Type of measurement | Systolic hypertension |
| --- | --- |
| OBPM | SBP ≥ 140 mmHg |
| 24H-ABPM | SBP ≥ 130 mmHg (day) |
| HBPM | SBP ≥ 135 mmHg |
| OBP30 | SBP ≥ 135 mmHg |

OBPM: office blood pressure measurement.

24H-ABPM: 24 hour ambulatory blood pressure measurement

HBPM: home blood pressure measurement

OBP30: office blood pressure measurement in 30 minutes

Ref.: Genootschap NH. NHG-standaard Cardiovasculair risico management (tweede herziening). Huisarts Wet. 2019;62(4):55-7.
